# Recent Advances in Transition-Metal Based Nanomaterials for Noninvasive Oncology Thermal Ablation and Imaging Diagnosis

**DOI:** 10.3389/fchem.2022.899321

**Published:** 2022-04-14

**Authors:** Qiuxia Peng, Zhangbo Qian, Huali Gao, Kun Zhang

**Affiliations:** ^1^ National Center for International Research of Bio-Targeting Theranostics, Guangxi Key Laboratory of Bio-Targeting Theranostics, Collaborative Innovation Center for Targeting Tumor Diagnosis and Therapy, Guangxi Talent Highland of Bio-Targeting Theranostics, Guangxi Medical University, Nanning, China; ^2^ Orthopedic Surgery Department, Institute of Arthritis Research in Integrative Medicine, Shanghai Academy of Traditional Chinese Medicine, Guanghua Hospital Affiliated to Shanghai University of Traditional Chinese Medicine, Shanghai, China; ^3^ Department of Medical Ultrasound and Central Laboratory, Shanghai Tenth People’s Hospital, Ultrasound Research and Education Institute, Tongji University School of Medicine, Shanghai, China

**Keywords:** transition-metal based nanomaterials, non-invasive thermal ablation, nanomedicine, imaging diagnosis, cancer

## Abstract

With the developments of nanobiotechnology and nanomedicine, non-invasive thermal ablation with fewer side effects than traditional tumor treatment methods has received extensive attention in tumor treatment. Non-invasive thermal ablation has the advantages of non-invasiveness and fewer side effects compared with traditional treatment methods. However, the clinical efficiency and biological safety are low, which limits their clinical application. Transition-metal based nanomaterials as contrast agents have aroused increasing interest due to its unique optical properties, low toxicity, and high potentials in tumor diagnosis. Transition-metal based nanomaterials have high conversion efficiency of converting light energy into heat energy, good near-infrared absorption characteristics, which also can targetedly deliver those loaded drugs to tumor tissue, thereby improving the therapeutic effect and reducing the damage to the surrounding normal tissues and organs. This article mainly reviews the synthesis of transition-metal based nanomaterials in recent years, and discussed their applications in tumor thermal ablation and diagnosis, hopefully guiding the development of new transition metal-based nanomaterials in enhancing thermal ablation.

## Introduction

Population growth and aging have led to high mortality and morbidity rates of cancer, becoming one of the main factors endangering physical and mental health (Bray et al., 2018). Tumors in humans result from the accumulation of mutations in cells’ DNA genes, disrupting the mechanisms that regulate cell division and cell death, resulting in the uncontrolled proliferation of dysfunctional cells (Alexandrov et al., 2013). Despite significant success in anti-tumor research, traditional treatment methods have limitations such as various adverse reactions, poor specificity and inducible drug resistance. Therefore, it is imperative to find more effective tumor treatment and diagnosis methods to reduce the economic burden of patients caused by cancer and improve their life quality. Recently, nanobiotechnology in the field of treating and diagnosing tumors have raised concerns, which is equipped with higher efficacy and safety than traditional treatment methods, holding great potentials in clinical transition (Shi et al., 2017).

Transition metals belong to groups 3–12 in the periodic table and are d-block elements with filled electron orbits, which can combine with other elements to form complex structures. Transition-metal based nanomaterials especially after combining metal atoms with surrounding anions or molecules (Xu M. et al., 2021) have received much attention due to their high photothermal conversion efficiency, low cytotoxicity, good photothermal stability, abundant elemental composition, and good biocompatibility (Zhao et al., 2018). They are more suitable as photo-thermal energy converters than molecular optical absorbers due to their plasmonic properties and high optical and thermal stabilities (Lapotko, 2009), making them promising candidates for tumor therapy and diagnosis.

Traditional tumor treatments such as chemotherapy, radiotherapy, and surgery still serve as the first-line treatment methods. However, they cause significant trauma to the body and fail to effectively prevent tumor recurrence and metastasis (Wang et al., 2020). Non-invasive thermal tumor ablation can selectively destroy multiple tumor foci, resulting in coagulative necrosis of tumor tissues, which is regarded to be a more attractive and logical treatment approach (Goldberg et al., 2000). In addition, tumor tissues are more sensitive to temperature than normal tissues (Chu and Dupuy, 2014). Currently, non-invasive thermal ablation techniques include photothermal ablation (PTA) (Zhang et al., 2019), radiofrequency ablation (RFA) (Zhang et al., 2016a; Zhang et al., 2016b; Fang et al., 2019), magnetothermal ablation (MHA), and high-intensity focused ultrasound (HIFU) (Zhang et al., 2014; Zhang et al., 2021). Recent researches have investigated the application of transition-metal based nanomaterials in nanomedicine (Chimene et al., 2015). Desirable characteristics such as high near-infrared (NIR) absorption and good thermal conductivity enable transition-metal based nanomaterials to be widely used in biosensors (Wang Y. H. et al., 2017), multimodal imaging, drug delivery (Jahangirian et al., 2019), PTA (Liu Y. et al., 2019), and MHA (Chang et al., 2021). These appealing properties adequately guarantee the high efficacy of non-invasive thermal ablation against tumors *via* increasing the ablation temperature and destroying tumor cells (Urbanova and Pumera, 2019).

**FIGURE 1:**
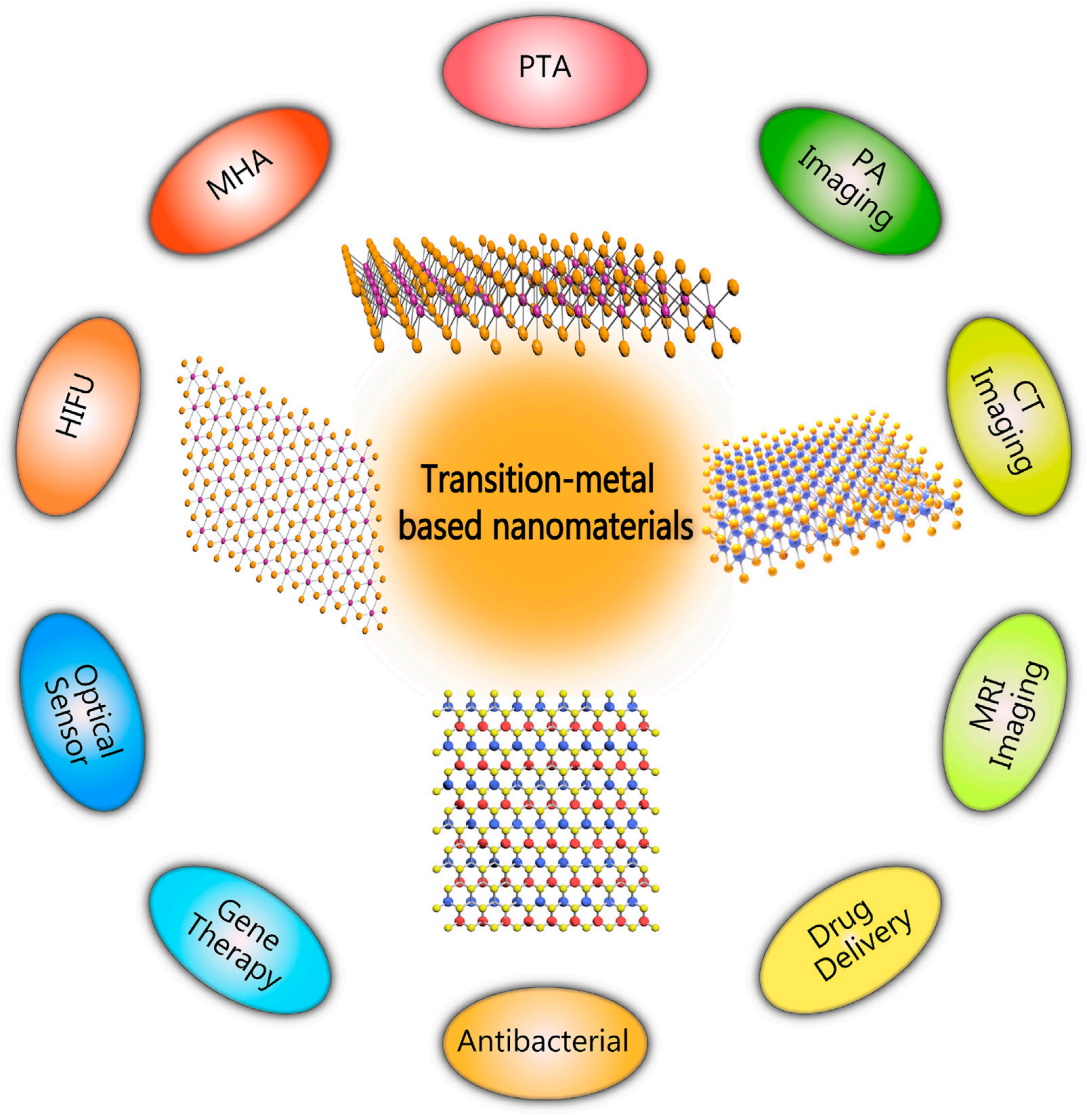
Schematic diagram of biomedical applications of transition-metal based nanomaterials.

This article mainly reviewed recent progress of transition-metal based nanomaterials in non-invasive thermal ablation of tumors, such as HIFU, PTA, and MHA (Figure 1). Their potential in improving energy conversion efficiency is emphasized, and their applications in imaging diagnosis was introduced. Finally, the prospects and development directions of transition-metal based nanomaterials were discussed.

## Transition-Metal Based Nanomaterials and Their Properties

**FIGURE 2:**
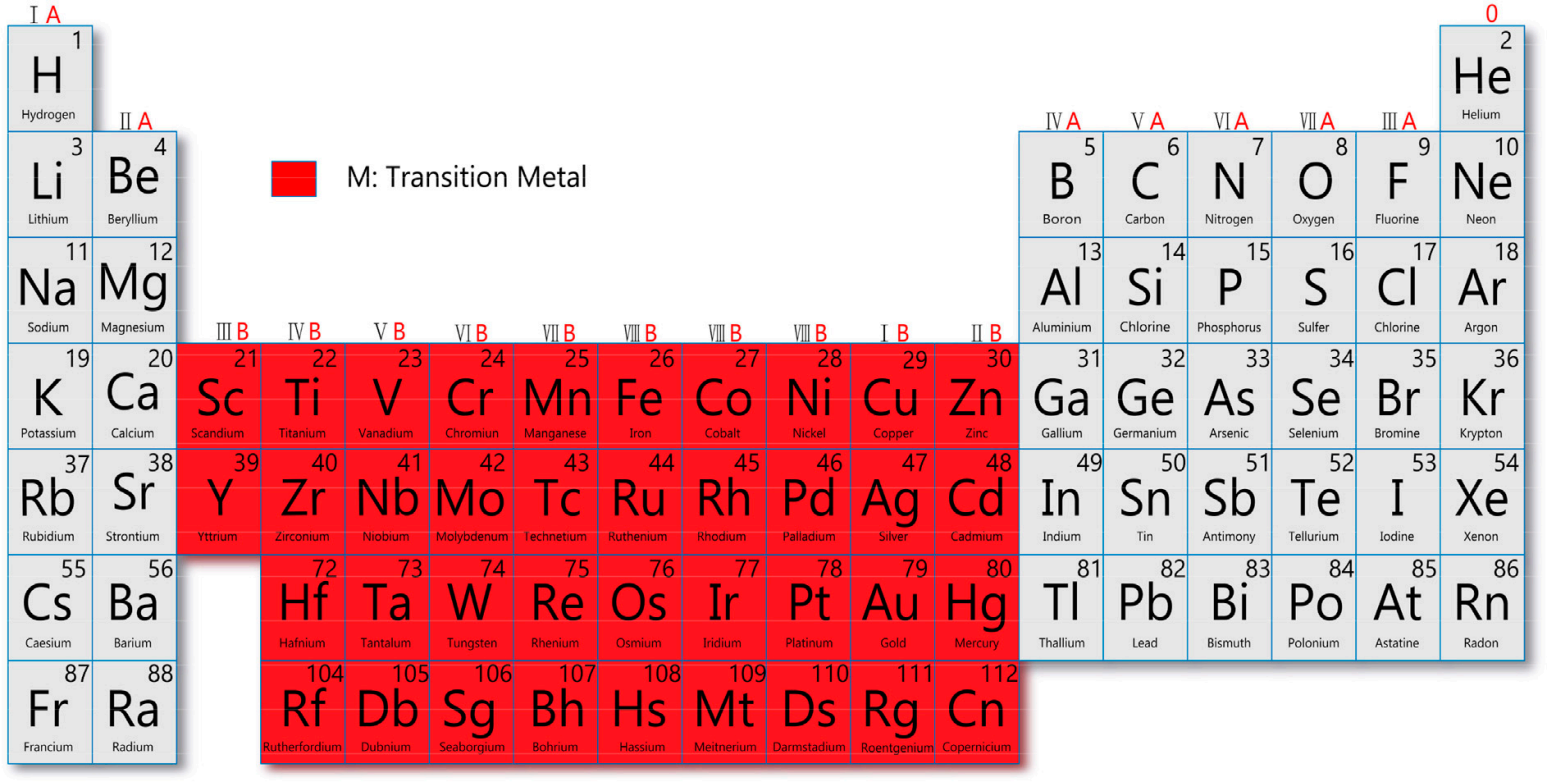
Schematic diagram of the distribution of transition metal elements.

Despite the successful application of cisplatin and aurein in clinics, transition-metal based nanomaterials have become a promising vehicle to deliver these drugs and have attracted much attention in the diagnosis and treatment of cancer (Luo et al., 2021). According to the definition by the International Union of Pure and Applied Chemistry, transition metals have atoms with incomplete d subshell, common cation, or free atom (Figure 2).

### Properties of Transition Metal Dichalcogenides

Transition Metal Dichalcogenides (TMDCs), denoted as MX_2_, are composed of transition metals; M denotes a group 4–7 element (such as Mo, W, Ta, Nb, and Mn), and X denotes chalcogens (such as S and Se) (Yun et al., 2020). The different structural compositions of TMDCs give them different properties, such as metals (NbS_2_, VSe_2_), semiconductors (MoS_2_, WS_2_), insulators (HfS), semi-metals (WTe_2_, TiSe_2_), and even superconductors (NbSe_2_, TaS_2_) (Zhang et al., 2018), for applications in different biological fields.

TMDCs have one layer of metal atoms sandwiched between two layers of chalcogen atoms. The van der Waals forces between the transition metals and sulfur atoms are weak. targeted delivery (Li et al., 2017). The high photothermal conversion efficiency (62.5%) of TMDC nanoparticles in the NIR region (650–900 nm) with strong absorption is of great significance for photoacoustic imaging (PA) and non-invasive tumor thermal ablation (Murugan et al., 2019). TMDCs have excellent optical and electrical properties, useful in various biosensors for detecting environmental pollution and bioactive molecules (Rohaizad et al., 2021). In addition, it was found that TMDCs can directly act on the cell wall of bacteria and destroy the vast majority of drug-resistant bacteria, potentially replacing antibiotics in the future (Debnath et al., 2021).

### Characteristics of Transition Metal Oxides

Transition metal oxide (TMO) nanomaterials are used in cancer treatment and diagnosis due to their unique composition, structure, and physicochemical properties (Wen et al., 2020). Transition metals are filled with electrons in the s orbital, while the d orbital is vacant. Hence, TMO nanomaterials have high dielectric constants, wide bandgaps, electronic transition, and excellent electrical properties (Jia et al., 2020), which is appropriate for engineering biosensors.

In terms of composition, TMOs can serve as oxidants because they contain oxygen. This allows TMOs to be reduced and decomposed into transition metal ions in an acidic, hypoxic tumor microenvironment with high levels of glutathione (Yang G. et al., 2021). Therefore, transition metal ions can be used for biological imaging, such as Mn ions-mediated magnetic resonance imaging (MRI) (Zheng et al., 2021). Oxygen in metal oxides can enhance the efficacy of photodynamic therapy (Lin T. et al., 2018) and sonodynamic therapy (SDT) (Xu Q. et al., 2021) of tumors.

### Characteristics of MXenes

MXenes are expressed as Mn+1XnTx, where M is a transition metal, and X is a carbon or nitrogen site; the maximum value of n is 4. Tx represents a functional group (VahidMohammadi et al., 2021). The functional group’s hydroxyl (OH), oxygen, or fluorine hydrophilicity is different from that of other transition-metal based nanomaterials. The advantages of MXenes, such as good biodegradability and biocompatibility, make it easier for use in nanomedicine (Lin H. et al., 2018). In addition, MXenes nanomaterials can be used as a delivery platform for anticancer drugs due to their large surface area, low toxicity, and targeting (Shukla, 2020). Photoacoustic imaging can acquire the unique optical properties of MXenes, and the excellent photothermal conversion efficiency determine that they can be used as a nanoreagent for PTT (Fu et al., 2021).

## Non-Invasive Thermal Ablation Assisted by Transition-Metal Based Nanomaterials

**FIGURE 3:**
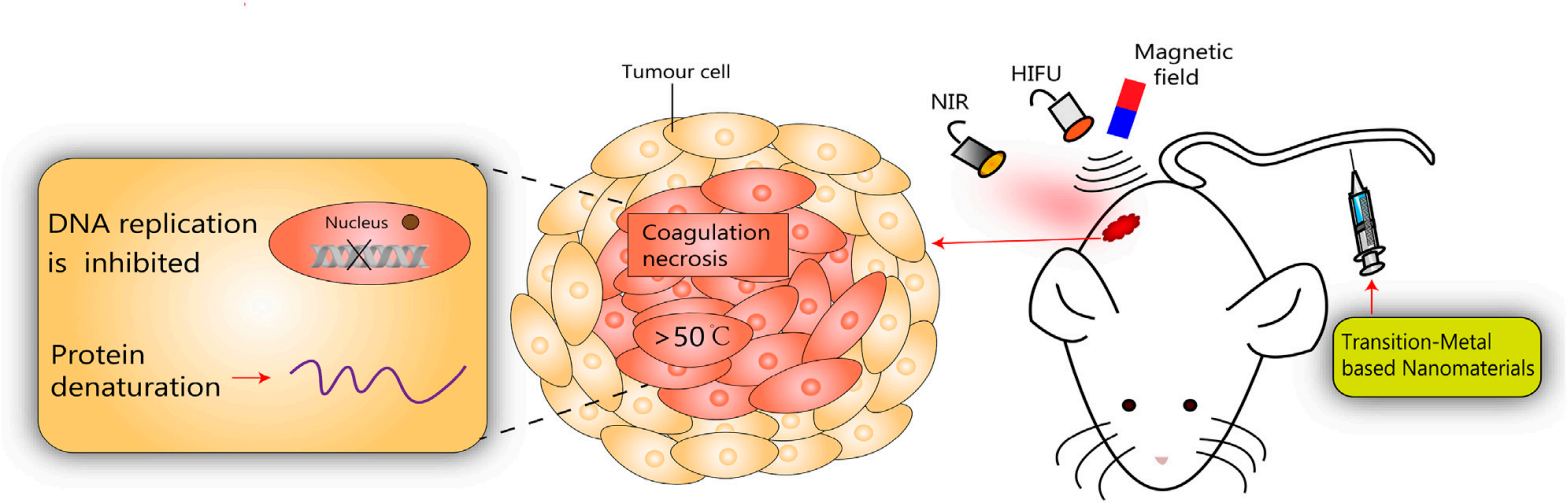
Schematic diagram of transition-metal based nanomaterials for noninvasive oncology thermal ablation.

Recently, thermal ablation therapy has emerged as a novel non-invasive treatment for localized solid malignancies by generating high temperature at the injury site, leading to protein denaturation, irreversible coagulative necrosis of tumor tissue, and rapid cell death (Wang M. et al., 2019). Noninvasive thermal tumor ablation modalities have been used clinically including PTA, MHA, and HIFU (Figure 3).

There are many similarities between the various thermal ablation methods. In a given lesion, the energy lost from heat plus the energy deposition from local tissue interactions equals the degree of coagulation necrosis (Goldberg et al., 2000). In recent years, attention has been paid to treat tumors using transition-metal based nanomaterials, and significant achievements have been made in auxiliary imaging diagnosis and cancer treatment. According to clinical demands, the rational design of transition-metal based nanomaterials can be endowed with special properties that increase the efficacy of thermal ablation of tumors. Some typical transition-metal based nanomaterials for non-invasive thermal ablation have been summarized in Table 1.

**TABLE 1:** Summary of transition-metal based nanomaterials for different thermal ablation methods.

Nanomaterial Type	Treatment method	References
Gold nanomaterials	PTA	Krzysztof et al. (2019)
Anti-MG1 HNP	PTA	White et al. (2017)
Gold nanorods	PTA	Yang et al. (2020)
CuS	PTA	Li et al. (2010)
F-CuS	PTA	Jang et al. (2018)
Gold-silica nanoshells	PTA	Rastinehad et al. (2019)
Gold nanocages	PTA	Zhou et al. (2019a)
Gold nanostars	PTA	Yuan et al. (2012)
Prussian blue	PTA	Xue et al. (2017)
PdMo bimetallene	PTA	Bai et al. (2021)
SPIO	MHA	Sadhukha et al. (2013)
MgA	MHA	Yang et al. (2021b)
FeNPs	MHA	Chao et al. (2019)
MAPP	HIFU	Wang et al. (2013)
PLGA-coated Fe_3_O_4_	HIFU	Sun et al. (2014)
Gold nanoparticle	HIFU	McLaughlan et al. (2017)
PFP-filled Fe-SiO_2_ nanoshells	HIFU	Liberman et al. (2014)

### Transition Metal-Based Nanomaterials-Assisted Photothermal Ablation

PTA treatment involves using optical absorbers, such as copper sulfide nanoparticles, gold nanostructures, and carbon nanomaterials, to generate heat under NIR laser irradiation. The resulting local hyperthermia can destroy diseased tissue cells, with little damage to natural tissues (Goncalves et al., 2020). PTA has significant advantages over traditional treatments, such as less invasiveness, strong cancer cell specificity, and rapid recovery (Ban et al., 2017). Transition-metal based nanomaterials as selective photothermal absorbers can improve the efficiency of PTA and reduce damage to surrounding tissues (Jaque et al., 2014). The non-invasive performance can be further improved by enhancing the photothermal conversion efficiency of photothermal treatment agents (PTTAs) (Doughty et al., 2019). PTTAs absorb light or energy and convert it into heat, inducing local hyperthermia that promotes tumor ablation (Yang et al., 2015). Compared with dye sensitizer molecules, transition-metal based nanoparticles have larger absorption cross-sections due to their strong surface plasmon resonance effect and higher photothermal conversion efficiency than other photothermal agents (Jain et al., 2006).

Gold nanomaterials (GNPs) are one of the widely used PTTAs due to their excellent photothermal conversion and ability to convert absorbed NIR light into heat to induce high local hyperthermia, low toxicity, and biocompatibility (Cheng et al., 2014; Xing et al., 2016; Gupta and Malviya, 2021). GNPs absorb incident photons and convert them into heat energy to increase the temperature locally, leading to cell death. GNPs can achieve high light absorption efficiency at lower radiant energy, ensuring high-efficient PTA (Krzysztof et al., 2019). White et al. constructed anti-MG1 conjugated hybrid magnetic gold nanoparticles. Their strong NIR absorption peak at 800 nm make it possible to target NIR PTA of tumors and proved to have a catalytic role in PTA, greatly improving tumor ablation efficiency (White et al., 2017). Gold nanorods (GNRs) are considered to be ideal photothermal sensors due to their inherently high biocompatibility, tunable localized surface plasmon resonance peaks, and versatile surface functionalization (Song et al., 2015). Yang et al. (2020) validated the excellent tumor ablation ability of GNRs under 980 nm illumination in a mouse xenograft model and demonstrated their photothermal therapy potential for tumors in the NIR window. CuS nanoparticles, nanobiomaterials for PTA of cancer, have the advantages of unique optical properties, low production cost and cytotoxicity, and small size (Goel et al., 2014). Li et al. (2010) synthesized CuS nanoparticles that can convert light into thermal energy and validated their strong absorption ability in the NIR region. In addition, CuS in NIR laser irradiation can greatly improve PTAs efficiency. Jang et al. (2018) constructed Fucoidan-coated copper sulfide nanoparticles to improve PTA rate effectively and verified their stable photothermal efficiency by measuring the UV-Vis absorption spectra before and after laser irradiation.

### Transition Metal-Based Nanomaterials-Assisted Magnetothermal Ablation

As a non-invasive local treatment strategy, MHA has received extensive attention in recent decades.

Because magnetic nanoparticles can absorb magnetic field energy, MHA can prevent unnecessary heating of surrounding healthy tissues, making it a promising tumor treatment modality (Thiesen and Jordan, 2008; Wang F. et al., 2017). The need for a magnetocaloric agent to induce heating effect, and the absence of tissue penetration limit in the used magnetic field, allows precise ablation of deep tumors (Yan et al., 2005). When the resistivity of the conductor is small (as in metals), the eddy currents induced by the alternating magnetic field (AMF) will be strong, and the resulting heat generated will be large. Therefore, transition-metal based nanomaterials make a strong, promising magnetic material, useful for magnetocaloric ablation (Muranaka et al., 2007).

The heat generated by iron peroxide nanoparticles (SPIO) in an oscillating radio frequency field is due to the hysteresis loss or Brownian rotation of the nanoparticles and depends on the oscillating magnetic field frequency. Under an applied electric field, the dipole interaction between adjacent particles increases the anisotropy; the concentration of nanoparticles can also lead to better heating performance (Chandrasekharan et al., 2020). Sadhukha et al. (2013) developed inhalable superparamagnetic iron oxide nanoparticles that were chelated with target-specific epidermal growth factor receptor for realizing targeted SPIOs accumulation in tumor, increasing thermal energy production and monitoring tumor ablation under MHT.

Many currently used magnetic nanoparticles, such as iron oxide, require strong AMFs for effective heating, while safer and more effective magnetocaloric formulations are required for tumor ablation therapy (Albarqi et al., 2019). Magnesium alloy (MgA) with excellent *in vivo* biocompatibility, biodegradability, and low elastic modulus, was widely used in clinical practice (Lin et al., 2019). Yang N. et al. (2021) verified that the MgA rods under magnetorheological fluid showed a significant temperature increase and MgA with a strong vortex thermal effect could effectively improve tumor PTA under a low magnetic field. Chao et al. (2019) found that pure iron nanoparticles (FeNPs) modified with polymers such as polyethylene glycol (PEG), stable in aqueous solution, can be used as an ultra-efficient magnetic material with sufficient heating under low-power AMF to achieve effective MHA. In addition, local injection of FeNPs-based nanomaterials for MHA can generate immune memory effect, inhibit tumor metastasis and prevent tumor recurrence.

### Transition Metal-Based Nanomaterial-Assisted High-Intensity Focused Ultrasound Ablation

HIFU that has been widely used in clinical practice can significantly reduce damages to the surrounding tissue with precise transfer of heat energy to the tumor site (Bachu et al., 2021). This needle-free, non-ionizing and thermal ablation tool has become a common method for non-invasive ablation of various solid tumors, harvesting inspiring clinical results (Wang M. et al., 2019). HIFU absorbs high acoustic energy and focuses it on the selected area, which raises the temperature in the tissue to above 60–65°C, resulting in protein denaturation and irreversible tumor tissue coagulation and necrosis (Diederich and Hynynen, 1999).

However, high-efficiency, high-power ultrasound treatment can burn surrounding skin and healthy tissue, leading to adverse effects. The application of ultrasound absorbers can enhance HIFU therapy (Dibaji et al., 2014). The photothermal conversion properties and high stability of Au nanomaterials (AuNPs) make them suitable for HIFU (Qian et al., 2017). In addition, AuNPs enhance the therapeutic effect of HIFU by increasing the temperature and sound energy absorption rate (Sadeghi-Goughari et al., 2019). Wang et al. (2013) synthesized MSNC@Au-PFH-PEG, abb. as MAPP, using AuNPs as the capping layer. They verified that the high thermal conductivity and thermal efficiency of finely anchored AuNPs significantly increase thermal energy accumulation to enhance the effect of tumor thermal ablation. In addition, MAPP is used in enhanced ultrasound imaging. Under the guidance, the ablation effect and accuracy of high-frequency ultrasound can be significantly improved.

The clinical use of HIFU is increasing, but damage to the skin and adjacent healthy tissues cannot be ignored. Superparamagnetic iron oxide nanoparticles (SPIONs) have great prospects in the biomedical field, especially as ultrasound absorbers, owing to their reliable sources and excellent superparamagnetic properties (Wei et al., 2021). Devarakonda et al. (as cited in Sun et al., 2014) improved the thermal ablation effect of tumors by combining the advantages of SPIONs and polymers to construct PLGA-coated Fe_3_O_4_ microcapsules to enable energy deposition and enhance the absorption of ultrasonic waves. In addition, PLGA-coated Fe_3_O_4_ microcapsules were used as MR-guided contrast agents for improving the accuracy of tumor localization while minimizing damage to surrounding normal tissues.

## Application of Transition-Metal Based Nanomaterials in Imaging Diagnosis

Because some tumors have no special symptoms in the early stages or are difficult to locate in the deep part of the body, most tumors are advanced or have metastases when diagnosed, with a low probability of cure. Therefore, early detection and diagnosis of cancer can give patients a chance for early cure and long-term survival. Currently used clinical imaging techniques, such as photoluminescence imaging, MRI, computed tomography (CT), positron emission tomography, ultrasound imaging, or optical imaging, hold great promise in cancer diagnosis (Huang et al., 2012). However, conventional contrast agents suffer from numerous problems, such as rapid bleaching, unsuitability for multicolor imaging, affected by the local chemical environment, interference from their background fluorescence, low brightness, and poor photostability (Sharma et al., 2006), limiting successful diagnosis. Depending on their good electrical conductivity, magnetic properties, biocompatibility, and non-toxicity (Sundaram et al., 2020), transition-metal-based nanomaterials can serve as contrast agents to build a nanomedicine platform for multimodal imaging-guided non-invasive thermal ablation such as photoacoustic imaging (Huang et al., 2018). The birth of this nanoplatform offers more opportunities for cancer patients to survive. We summarized some bioimaging applications of transition-metal based nanomaterials in Table 2.

**TABLE 2:** Summary of bioimaging applications of transition-metal based nanomaterials.

Transition-metal based nanomaterials	Bioimaging modality	References
AlPc-MoS_2_@SiO_2_-CS	CT / PA/NIRF	Liu et al. (2018a)
CMPB-MoS_2_-PEG	MRI	Guan et al. (2022)
LDH-MoS_2_ (LMM)@BSA	MRI	Zhao et al. (2021)
HA-MoS_2_	PA/FL	Shin et al. (2019)
MoS_2_@ss-SiO_2_	FL/CT/MSOT	Li et al. (2019)
MoS_2_-Au(MA)-PEG	CT/PA	Liu et al. (2019a)
MSNR@MoS_2_-HSA/Ce6	CT/FL/ MOST	Yang et al. (2019)
MoS_2_–CuO@BSA/R837 (MCBR)	CT/IR/MRI	Jiang et al. (2021)
MoSe_2_(Gd3+)-PEG	PA/MRI	Pan et al. (2018)
Bi_2_Se_3_/MoSe_2_(Bi-M-3)@PEG-Dox	CT/PT	Wang et al. (2019d)
MPDA-WS_2_@MnO_2_	CT/MRI/MOST	Wang et al. (2019c)
WLPD-Au25	CT/NIRF	Zhou et al. (2019b)
WID@M-FA	PA/NIRF	Long et al. (2020)
WS_2_-IO/S@MO-PEG	PA/MRI	Yang et al. (2018)
ReS_2_	CT	Wang et al. (2019b)
TSIO	PA/MRI	Fu et et al. (2020)
Cu_2_MnS_2_	MRI/MSOT	Ke et al. (2017)
HPFeS_2_@C-TA-PEI-GOx-FA	US/PA/MRI	Wu et al. (2020)
CFMS-PVP	PA	Zhu et al. (2020)
Gd/CuS@PEI-FA-PS	PA/MRI	Zhang et al. (2020)
Fe_3_O_4_@MnO_2_–Ce6/CSL	FL/PA/MRI	Fan et al. (2021)
UCCM	CT/MRI/UCL	Ni et al. (2022)
MnO_2_/Ag_3_SbS_3_	PA/MRI	Wang et al. (2022)
WTO-PEG	PA/CT	Gao et al. (2019)
B-TiO_2_@SiO_2_–HA	PA	Guo et al. (2021)
Fe(II)-Ti_3_C_2_ (FTC)	MRI	Wu et al. (2021)
Fe_3_O_4_/MnOx–Nb_2_C-SP	MRI	Liu et al. (2022)
Ti_3_C_2_Tx-Pt-PEG	PA	Zhu et al. (2022)
Ta_4_C_3_-IONP-SPs	CT/MRI	Liu et al. (2018b)
Ti_3_C_2_@Au	PA/CT	Tang et al. (2019)

### Bioimaging Applications of Transition Metal Dichalcogenides

We mainly introduce the latest TMDC composite nanomaterials for bioimaging applications. Tungsten disulfide (WS_2_), Molybdenum disulfide (MoS_2_), and titanium disulfide (TiS_2_) received more attentions in bioimaging with the advantages of catalytic performance, photoluminescence, light absorption, and high wear resistance (Rohaizad et al., 2021). Liu L. et al. (2018) synthesized a novel chitosan (CS)-controlled aluminum chloride phthalocyanine (AlPc)-supported MoS_2_ nanocomposite (AlPcMoS_2_@SiO_2_-CS) with high HU value of 12HU Lg^−1^. This nanocomposite can solve the defects of short circulating half-life and non-specific distribution of traditional CT contrast agents and overcome poor tissue penetration and low sensitivity of PA (Liu et al., 2015). With the 4T1 mouse tumor-bearing model, they intravenously injected AlPcMoS_2_@SiO_2_-CS into mice and recognized a strong PA signal at tumor. Therefore, AlPc-MoS_2_@SiO_2_-CS can perform CT/PA dual-modal imaging, which is a non-invasive method for tumors.

**FIGURE 4:**
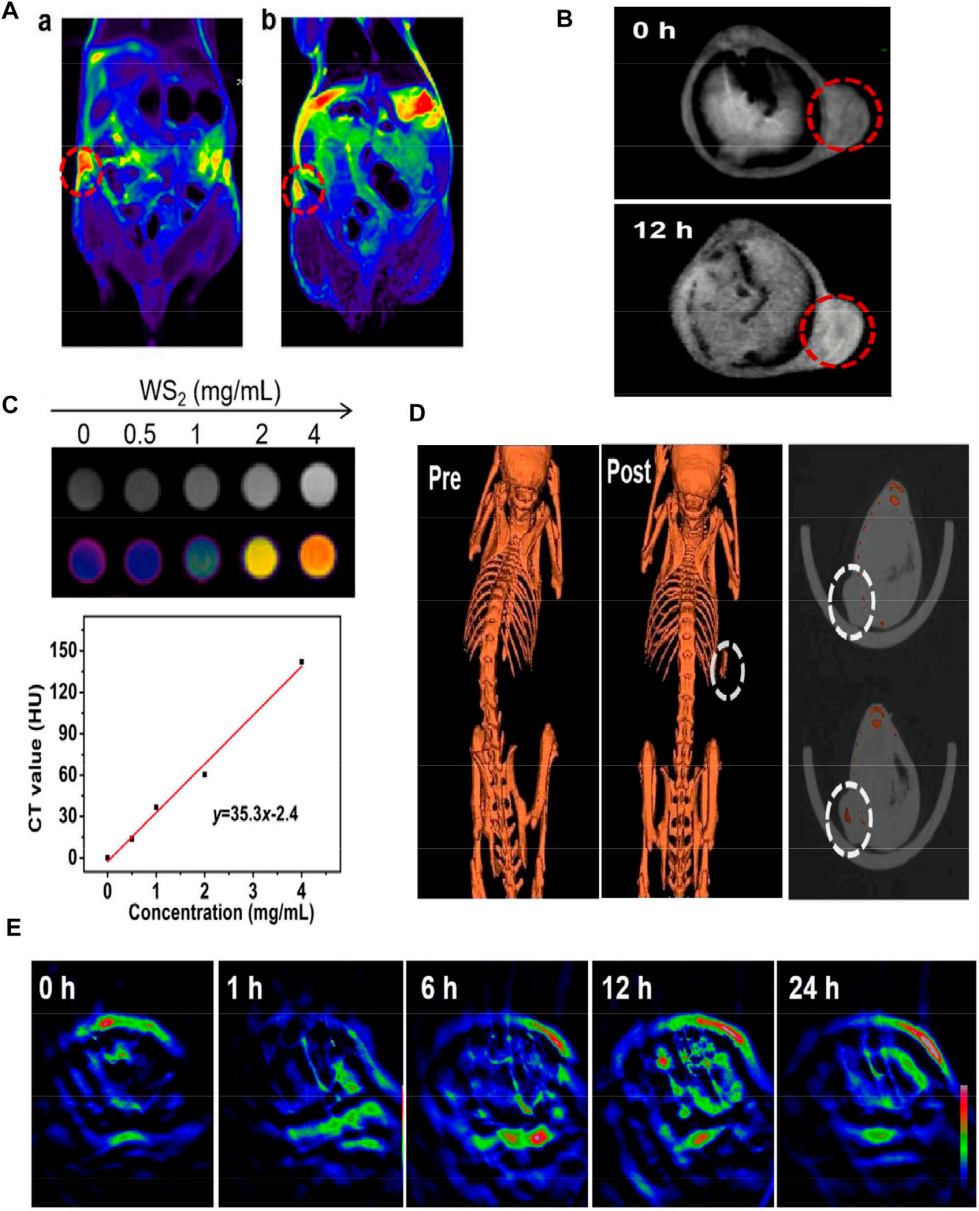
**(A)** T2-weighted MRI before and after injection of CMPB-MoS_2_-PEG. **(B)** T1-weighted MRI before and after injection of MPDA-WS_2_@MnO_2_. **(C)** The CT value (Hounsfield units, Hu) increased linearly with the increase of the concentration. **(D)** CT imaging of tumor on mice before and after injection of MPDA-WS_2_@MnO_2_. **(E)** MOST imaging of tumor on mice before and after injection of MPDA-WS_2_@MnO_2_. Reprinted (adapted) with permission from Guan et al. (2022).J Colloid Interface Sci. 2022, 608 (Pt 1),344–354. Copyright 2021 Elsevier Inc. Reprinted (adapted) with permission from Wang et al. (2019c). biomaterials. 2019,220,119405.Copyright 2019 Elsevier Ltd.

MRI is a non-invasive imaging technique that can distinguish image contrast difference between normal and diseased tissues (Iima and Bihan, 2016). Nowadays, people use Prussian blue containing ferric ions as a T2-weighted MRI agent to guide MHA (Zhu et al., 2015). Shao et al. (as cited in Guan et al., 2022) synthesized a transition metal composite nanosheet (CMPB-MoS_2_-PEG) composed of Cu/Mn ion-doped Pb and MoS2 for MRI of tumors and can aggregate at tumor sites for a long time (Figure 4A). Zhao et al. (2021) used layered double hydroxide and bovine serum albumin (BSA)-modified MoS_2_ to obtain a composite transition-metal based nanomaterials for T1-weighted MRI. Utilizing the characteristics of the tumor microenvironment, hypoxia, weak acid, reducing (Watson et al., 2021), intravenous injection of LMM@BSA clay sheets into H29 cell tumor-bearing mice showed the significantly-enhanced *in vivo* MR imaging brightness.

Multispectral photoacoustic tomography is an emerging imaging technique that combines the advantages of optical and ultrasound imaging with high resolution and sensitivity (Ke et al., 2017). Wang et al. (2019c) synthesized mesoporous dopamine (MPDA) and manganese dioxide (MnO_2_) onto WS_2_ to obtain MPDA-WS_2_@MnO_2_ nanoparticles. In 4T1 tumor-bearing mice, CT signal increased significantly from 52 HU Lg^−1^ to 419 HU Lg^−1^, 30 min after administration, while the PA signal and T1-weighted signal doubled after 12 h (Figure 4B). This CT/MOST/MR multi-modal imaging makes transition-metal based nanomaterials more accurate and reliable in biological imaging (Figures 4C–E). Fu et al. (2020) anchored iron oxide (IO) on titanium disulfide (TiS_2_) nanosheets to obtain TISO nanoplatforms as T2-weighted contrast agents for MRI, increasing the T2 relaxation rate (R2) by 8.9 times. In addition, the NIR window (NIR-II) irradiation increased the PA imaging amplitude by 1.58 times, and a clearer MR/PA image was obtained.

### Transition Metal Oxides in Bioimaging Applications

Compared with TMDCs, TMOs have redox and cation exchange capabilities (Jia et al., 2020). Currently, their research is still at a relatively nascent stage, so we will elaborate on the application of MnO_2_ and TiO_2_ in bioimaging, the hottest research in recent years. Fan et al. (2021) successfully prepared Fe_3_O_4_@MnO_2_–Ce6/CSL nanodiagnostic platform by loading terpenoid (CSL)/photosensitizer Ce6 on the surface to grow Fe_3_O_4_ MnO_2_ nanoparticles. In Bel-7402 tumor-bearing nude mice, the Mn and Fe ions released by the nanomaterials were found to enhance the magnetic resonance T1 signal and weaken the T2 signal. Moreover, the release of Ce6 can achieve fluorescence imaging (FL) and a strong PA signal. Thus, this triple imaging platform of MRI/FL/PA can clearly locate the tumor. Gao et al. (2019) showed that the HU value of WTO nanoparticles obtained by PEGylation of W-doped TiO_2_ was 7.9 HU Lg^−1^ higher than the 6.3 HU Lg^−1^ using the traditional contrast agent (iopromide), so the CT signal become stronger. In addition, the strong absorption in the NIR-II window makes it possible to use glycated WTO for dual-mode imaging of CT/PA.

### MXenes in Bioimaging Applications

MXenes are 2D carbides, nitrides, and carbonitrides representing transition metals. The most distinctive feature of MXenes is their ability to target tumor cells with minimal cytotoxicity to nonmalignant cells (Szuplewska et al., 2020). They are also highly conductive and magnetic, making them a diagnostic tool for cancer (VahidMohammadi et al., 2021). Wu et al. (2021) anchored ferrous ions on the Ti_3_C_2_ nanolayer to obtain a multifunctional nanoplatform of Fe(II)-Ti_3_C_2_ (FTC). They found that the MRI imaging time in MKN45 tumor-bearing nude mice was as long as 24 h, creating a time window for treating tumors. Liu et al. (2022) successfully synthesized Fe_3_O_4_/MnOx–Nb_2_C-SP composite MXenes nanoplatform by loading magnetic Fe_3_O_4_/MnOx on niobium carbide (Nb_2_C) ultrathin nanosheets and modifying them with soybean phospholipid. The platform injected 4T1 tumor-bearing mice intravenously, and the results showed that the T1 signal intensity increased by 43.21%, while the T2 signal intensity decreased by 31.43%, achieving T1/T2 contrast-enhanced MRI imaging.

## Conclusion and Outlook

Nanotechnology is a new research field that has developed rapidly in recent years. It has had a huge impact in multiple research fields while simultaneously providing significant challenges and opportunities. A large number of studies have shown that transition-metal based nanomaterials can not only effectively improve the tumor treatment effect of various ablation techniques through various mechanisms, but also provide the possibility for early diagnosis and treatment of tumors, and more importantly, inhibit tumor metastasis and prevent tumor recurrence. Transition-metal based nanomaterials are important in medicine and pharmaceutical sciences, mainly because of their combined catalytic and redox properties and coordination abilities. In this paper, the characteristics and properties of transition-metal based nanomaterials are reviewed. Transition-metal based nanomaterials provide accurate and efficient tumor thermal ablation, while biological imaging can be used for early and differential diagnosis of diseases.

Despite the attractive results of transition-metal based nanomaterials, there are still some issues that need to be addressed. First, the current research on transition-metal based nanomaterials is limited, and the systematic biosafety is not clear enough; secondly, these nanomaterials lack sufficient targeting ability and may cause damages to normal human tissues or cells. Although transition-metal based nanomaterials have many problems to be solved, they still provide unlimited potential and great chances for cancer treatment and diagnosis, and their research progress opened up new fields in diagnosing and treating many diseases.
